# Ambulatory consolidation chemotherapy for acute myeloid leukemia with antibacterial prophylaxis is associated with frequent bacteremia and the emergence of fluoroquinolone resistant E. Coli

**DOI:** 10.1186/1471-2334-13-284

**Published:** 2013-06-22

**Authors:** Lalit Saini, Coleman Rostein, Eshetu G Atenafu, Joseph M Brandwein

**Affiliations:** 1 Department of Medical Oncology and Hematology, Princess Margaret Hospital, University of Toronto, 610 University Avenue, Rm. 5-109, Toronto, ON M5G 2M9, Canada; 2 Division of Infectious Diseases, University Health Network, Toronto, ON, Canada; 3 Department of Biostatistics, Princess Margaret Hospital, University of Toronto, 610 University Avenue, Rm. 5-109, Toronto, ON M5G 2M9, Canada

**Keywords:** Acute myeloid leukemia, Chemotherapy, Infections, Bacteremia, Antibiotic resistance

## Abstract

**Background:**

Ambulatory consolidation chemotherapy for acute myeloid leukemia (AML) is frequently associated with bloodstream infections but the spectrum of bacterial pathogens in this setting has not been well-described.

**Methods:**

We evaluated the emergence of bacteremias and their respective antibiotic susceptibility patterns in AML patients receiving ambulatory-based consolidation therapy. Following achievement of complete remission, 207 patients received the first cycle (C1), and 195 the second cycle (C2), of consolidation on an ambulatory basis. Antimicrobial prophylaxis consisted of ciprofloxacin, amoxicillin and fluconazole.

**Results:**

There were significantly more positive blood cultures for *E. coli* in C2 as compared to C1 (10 vs. 1, p=0.0045); all *E. coli* strains for which susceptibility testing was performed demonstrated resistance to ciprofloxacin. In patients under age 60 there was a significantly higher rate of *Streptococccus spp*. bacteremia in C2 vs. C1; despite amoxicillin prophylaxis all *Streptococcus* isolates in C2 were sensitive to penicillin. Patients with *Staphylococcus* bacteremia in C1 had significantly higher rates of *Staphylococcus* bacteremia in C2 (p=0.009, OR=8.6).

**Conclusions:**

For AML patients undergoing outpatient-based intensive consolidation chemotherapy with antibiotic prophylaxis, the second cycle is associated with higher rates of ciprofloxacin resistant *E. coli*, penicillin-sensitive *Streptococcus* bacteremias and recurrent *Staphylococcus* infections.

## Background

Intensive treatment for acute myeloid leukemia (AML) typically includes induction followed by 2–4 cycles of consolidation chemotherapy [1]. In recent years there has been a shift to outpatient-based AML consolidation therapy with a number of studies confirming the safety of this approach for selected patients [2-9].

In most centers ambulatory chemotherapy programs rely on the use of antimicrobial prophylaxis as a means of reducing infections. Recent Infectious Disease Society of America (IDSA) guidelines endorse the use of ciprofloxacin prophylaxis to reduce bacterial infections in high risk cancer patients receiving chemotherapy [10]. Despite prophylactic antibiotics 30-90% of ambulatory patients experience a fever and 20-90% of consolidation cycles are associated with hospitalizations [2,4-6]. However, there are limited data on the characteristics of infectious complications in this setting. We have previously shown that infectious complications increase in the second cycle of ambulatory AML consolidation therapy resulting in greater need for intensive care unit (ICU) support and increased mortality [11]. As these findings may relate to breakthrough infections caused by the development of antibiotic resistant microbes despite prophylactic antibiotic use, we conducted a retrospective analysis evaluating the spectrum of microbiological isolates and their antibiotic resistance patterns in AML patients undergoing ambulatory-based consolidation chemotherapy.

## Methods

### Patients

All newly diagnosed AML patients at the Princess Margaret Hospital (PMH) who had achieved complete remission with frontline induction chemotherapy and proceeding to ambulatory-based consolidation chemotherapy from October 2002 - February 2008 were included in the analysis. Eligibility requirements were as previously described [11]. Patients received the second consolidation cycle following peripheral count recovery from the first cycle and resolution of any previous bacterial infections. Prior approval for the study was obtained from the Cancer Registry Data Access Committee and Research Ethics Board of the University Health Network, Toronto, Ontario.

### Chemotherapy regimens and supportive care

Ambulatory chemotherapy, subsequent monitoring and supportive care were provided as previously described [11] either in the ambulatory care area of the PMH or a local community hospital/clinic. Patients under age 60 (younger cohort) received two cycles of consolidation therapy (C1 and C2), each consisting of high dose cytarabine (HiDAC) 3 grams/m^2^ IV q12 hours × 6 doses on days 1, 3, and 5, plus daunorubicn 45 mg/m^2^ IV daily on days 1 and 2. For patients over age 60 (older cohort), C1 consisted of daunorubicin 60 mg/m^2^ IV daily × 3 plus cytarabine 100 mg/m^2^ as a 7-day continuous infusion via ambulatory infusion pump and C2 consisted of mitoxantrone 10 mg/m^2^ IV plus etoposide 100 mg/m^2^ IV, each given once daily from days 1–5.

During induction therapy patients received antimicrobial prophylaxis with fluconazole 400 mg PO daily whereas during consolidation they received ciprofloxacin 500 mg PO q12 hours, amoxicillin 500 mg PO q8 hours and fluconazole 400 mg PO daily, starting on Day 8 of the chemotherapy cycle and continuing until absolute neutrophil count (ANC) > 0.5 × 10^9^/L. All chemotherapy, transfusions and IV antibiotics were administered via central venous catheters (CVC), usually double lumen Hickman lines placed prior to the start of induction chemotherapy.

Patients presenting with fever or clinical features of an infection had blood cultures drawn from all CVC ports, a peripheral vein and any other accessible sites suspected of being infected. As per institutional policy, empiric antibiotics for febrile neutropenia most commonly consisted of pipracillin/tazobactam 4.5 grams IV q8 hours plus tobramycin 5 mg/kg IV q24 hours. Febrile patients with suspected CVC involvement i.e. those with erythema, pain or purulent drainage at the insertion site, were started on vancomycin 1 gram IV q12 hours. Empiric therapy was adjusted based upon the results of culture and sensitivity testing. Antimicrobials were continued until microbiological and clinical evidence of infection had resolved and the ANC was > 0.5 × 10^9^/L. Hematopoietic growth factors were not routinely used but were administered at the physician’s discretion from day 16–18 until neutrophil recovery. Patients with blood cultures positive for coagulase negative staphylococci (CNST) usually retained their CVC whereas patients with candidemia, tunnel infections or those with persistently positive cultures beyond 72 hours had their CVC replaced.

Cultures were processed using standard microbiological methods. Antibiotic susceptibilities were performed and reported by the Department of Microbiology of the Mount Sinai Hospital/University Health Network (Toronto, Ontario) or the local community hospital (if the site of the patient’s initial presentation) using predefined criteria. Antibiotic sensitivity data were not available in some cases where patients presented to local community hospitals.

### Definitions

Blood cultures were considered positive if one or more culture bottles showed growth, regardless of the organism, with symptoms compatible with an infection. Bacteremias related to common contaminants such as CNST, *Micrococcus*., *Propionibacterium* or *Bacillus* spp. were treated with a full course of antibiotics; thus no distinction was made between contaminants and true pathogens. Cultures were considered polymicrobial if at least two different organisms were isolated. Repeated cultures in persistently febrile patients yielding the same organism with the same antibiotic susceptibility profile were considered as one isolate unless separated by one or more negative blood cultures.

### Statistical analysis

Descriptive statistics were used to describe patient characteristics and outcome recordings. Categorical variables were expressed as count and proportions whereas continuous variables were expressed as means. Pearson Chi-square or Fisher’s exact test, when applicable, were used when comparing rates of infection and readmission. Results were considered significant if two-tailed P was < 0.05. Statistical Analysis Software (SAS, version 9.2; SAS Institute Inc., Cary NC) was used for statistical analysis.

## Results

### Patient characteristics

The characteristics of the patients receiving ambulatory-based consolidation chemotherapy have been previously described [11], and are summarized in Table 1. There was no significant difference in the median age of patients between C1 and C2; 54.6% of patients in C1 and 52.3% in C2 were male and the proportion of patients age ≥ 60 was 30% in C1 and 33% in C2 (p=NS).

**Table 1:** Blood culture results

	**C#1**	**C#2**	**(p-value)**
**Total Number of Patients**	196	187	
Age < 60	140	126	
Age ≥ 60	56	61	
% Male	107 (54.6%)	98 (52.3%)	
At least one blood culture positive (% of patients)	43 (21.9%)	59 (31.6%)	**P=0.03**
1 culture positive	37 (86%)	53 (90%)	NS
2 or more cultures positive	6 (14%)	6 (10%)	NS
Patients with Gram Positive isolates (% of Patients)	35 (17.9%)	46 (24.6%)	0.11
Patients with Gram Negative isolates (% of patients)	11 (5.6%)	20 (10.7%)	0.07
**Gram Positive vs. Gram Negative**	**P=0.0002**	**P=0.0004**	
Total number of positive blood cultures:	49	65	
Monomicrobial cultures (% of total cultures)	45 (91.8%)	60 (92.3%)	NS
Polymicrobial cultures (% of total cultures)	4 (8.2%)	5 (7.7%)	NS
Total number of isolates:	53	71	
Age < 60	40	44	
Age ≥ 60	13	27	
**Clinical Isolates (% of patients)**			
**Gram Positive**^**+**^	35 (17.9%)	46 (24.6%)	P=0.10
CNST*	19 (9.7%)	23 (12.4%)	NS
*Corynebacterium*	5 (2.6%)	2 (1.1%)	
*Micrococcus*	4 (2.0%)		
*Enterococcus*	1 (0.5%)	3 (1.6%)	
*Staphyloccoccus. aureus*	2 (1.0%)	1 (0.5%)	
***Streptococcus species***	**9 (4.6%)**	**16 (8.6%)**	**P=0.11**
*Streptococcus viridins*	6 (3.1%)	13 (7.0%)	
*Streptococcus viridians/Streptococcus oralis*	-	1 (0.5%)	
*Streptococcus mitis*	2 (1.0%)	-	
*Streptococcus auralis*	-	1 (0.5%)	
*Streptococcus spp.*	1 (0.5%)	1 (0.5%)	
< age 60 with Strep.	6 (4.3%)	14 (11.2%)	**P=0.033**
≥ age 60 with Strep.	3 (5.36%)	2 (3.3%)	NS
Other Gram Positive	1 (0.5%)	4 (2.1%)	
**Gram Negative**	11 (5.6%)	20 (10.7%)	P=0.07
Age < 60	8 (5.7%)	9 (7.1%)	**NS**
Age ≥ 60	3 (5.4%)	11 (18%)	**P=0.035**
*Eschericia coli*	1 (0.5%)	10 (5.3%)	**P=0.005**
*Klebsiella*	2 (1.0%)	5 (2.7%)	
*Enterobacter*	3 (1.5%)	1 (0.5%)	
*Pseudomonas spp.*	4 (2.0%)	-	
Other Gram Negative	1 (0.5%)	4 (2.1%)	
**Yeast**	-	1 (0.5%)	

CNST* = coagulase-negative staphylococci;

Gram Positive^+^ = Species information available for 185 pts. in C2 (Age < 60=125, Age ≥ 60=65).

### Bacteremia during ambulatory chemotherapy

Microbiologically documented episodes of bacteremia are shown in Table 1. As we previously reported, the ambulatory cohort in C2 had significantly higher rates of positive blood cultures and bloodstream infection relative to C1 [11]. Most patients had only a single positive blood culture (86% in C1 and 90% in C2) with greater than 90% of cultures in each cycle being monomicrobial. In each consolidation cycle significantly more patients had a gram-positive bacterial infection as compared to gram-negative infections. The proportion of gram positive vs. gram negative isolates was not significantly different in C1 as compared to C2. Approximately 30% of patients presented to local community hospitals, where the source of the positive blood culture (CVC vs. peripheral) was not identified; we were therefore unable to determine the extent of CVC related bacteremias in these cases.

As shown in Table 1, CNST were the most common gram positive isolates during both cycles. *Streptococci* constituted 17% (9/53) of the total isolates in C1 and 23.9% (17/71) of the total isolates in C2 (p=0.35). Younger patients had a significantly higher incidence of streptococcal bacteremia in C2 relative to C1 (11.2% vs. 4.3%, p=0.033), and in these patients *streptococci* represented a greater proportion of the total isolates in C2 (34%, 15/44 isolates) relative to C1 (15%, 6/40 isolates, p=0.044).

Gram negative organisms were identified in 5.6% of patients in C1 vs. 10.7% of those in C2 (p=0.07). The frequency of *E. coli* isolates was significantly higher in C2 relative to C1 (5.4% vs. 0.5%, p=0.005) with *E. coli* constituting 14.1% (10/71) of all isolates in C2 and 1.9% (1/53) of those in C1 (p=0.023). This trend was observed in both the younger (0 in C1 vs. 4 in C2) and older (1 in C1 vs. 6 in C2) cohorts. In addition, there was no difference in the incidence of *E. coli* bacteremia in younger versus older patients within C1 (p=0.827) or C2 (p=0.8). The proportion of patients with non-*E. coli* gram negative isolates did not differ between C1 and C2 (5.1% vs. 5.3%, p=0.91).

### Antibiotic resistance patterns

Table 2 highlights the antibiotic resistance patterns for selected organisms. Only one of the CNST with antibiotic sensitivity data was resistant to vancomycin while virtually all isolates were resistant to cloxacillin. All Streptococcus spp. isolates with susceptibility data exhibited sensitivity to penicillin. Among the non- *E. coli* gram-negative isolates, there was no difference in rates of ciprofloxacin resistance between C1 and C2 (1/9 vs. 0/7, p=1.0). In contrast, all *E. coli* isolates (8/8) from patients in C2 for which susceptibilities were available were ciprofloxacin resistant.

**Table 2:** Antibiotic resistance patterns of isolates (number showing resistance / isolates with antibiotic sensitivity data available)

	**Consolidation 1**	**Consolidation 2**
	**(% of isolates)**	**(% of isolates)**
CNST	20 (37.7%)	23 (32.4%)
**Vancomycin resistant**	**1 / 15**	**0 / 21**
Cloxacillin resistant	14 / 17	21 / 22
*Streptococcus species*	9 (17%)	17 (23.9%)
Penicillin resistant	1 / 7	0 / 11
Vancomycin resistant	0 / 3	0 / 6
Gram Negatives	11 (20.7%)	20 (28.1%)
**Ciprofloxacin resistant**	**2 / 10**	**8 / 15**
P/T* resistant	1 / 10	0 / 14
Ampicillin resistant	9 / 9	13 / 15
Tobramycin resistant	1 / 8	3 / 10
*Klebsiella species*	2 (3.8%)	5 (7.0%)
Ciprofloxacin resistant	0/2	0 / 4
P/T* resistant	0/2	0 / 4
Ampicillin resistant	2/2	4 / 4
Tobramycin resistant	0/2	0 / 3
*Escherichia coli*	1 (1.9%)	10 (14.1%)
**Ciprofloxacin resistant**	**1/1**	**8 / 8**
P/T* resistant	0 / 1	0 / 8
Ampicillin resistant	1 / 1	7 / 8
Tobramycin resistant	1/1	3 / 5
*Pseudomonas species*	4 (7.6%)	-
Ciprofloxacin resistant	1 / 4	-
P/T* resistant	1/1	-
Ampicillin resistant	3/3	-
Tobramycin resistant	0 / 4	-

*P/T = Piperacillin/tazobactam.

As shown in Table 2, most gram negative isolates in C1 and C2 were sensitive to the commonly used empiric antibiotics for febrile neutropenia in our institution, piperacillin/tazobactam and aminoglycosides. However, tobramycin resistance was noted in 67% (4/6) of the *E. coli* isolates, including 3/5 isolates from patients in C2.

### Infection-related mortality

Overall, 9 patients died due to infectious complications while receiving ambulatory chemotherapy, one patient during C1 and 8 during C2. CNST were isolated from one such patient during C2 whereas *Streptococcus spp.* was isolated from two patients who died (one each in C1 and C2). The single *Streptococcus* isolate with available antibiotic susceptibility information was sensitive to penicillin and vancomycin. Gram negative organisms were isolated in 6 of the 8 patient deaths in C2: *Klebsiella* spp*.* (3 cases), *E. coli* (2) and *Pseudomonas* spp*.* (1, urine isolate). Both *E. coli* isolates were resistant to ciprofloxacin and tobramycin and displayed sensitivity only to piperacillin/tazobactam. Mortality amongst patients with ciprofloxacin resistant gram-negative bacteremias was 20% (2/10) versus 13.3% (2/15) for those with ciprofloxacin sensitive strains (p=1.0).

### Effect of previous positive cultures/hospitalization

Approximately 51% of patients with a positive blood culture in C1 had a subsequent positive culture in C2 while only 27% of those with negative blood cultures in C1 had a positive culture in C2 (OR=2.8, p=0.005). This trend was significant for gram-positive organisms (OR=3.25, p=0.004) but not for gram-negative organisms (OR=2.1, p=0.31). An infection with *Staphylococcal* isolates in C1 was associated with the highest recurrence rates in C2 but only for patients age <60 (OR=8.6, p=0.009). No such association was seen for *Streptococcal* isolates or in the older cohort.

## Discussion

Recent data suggest that ambulatory chemotherapy for AML patients can reduce the incidence of septicemia [7]. However, published data on the characteristics of microbiological isolates from patients receiving ambulatory chemotherapy remains limited [2-7,9]. Our study, focusing on bacterial bloodstream infections, represents the largest reported series on AML patients receiving ambulatory-based intensive consolidation chemotherapy. We have previously shown that the rate of positive blood cultures increases following the second consolidation cycle [11] and the current study highlights potential explanations for the observed increase.

Despite the isolation of ciprofloxacin resistant *E. coli* in febrile neutropenic patients receiving fluoroquinolone prophylaxis [7,12,13] their use in high risk patients receiving chemotherapy has been supported by large randomized clinical trials and meta-analysis [14-17]. Within our cohort we observed an increased incidence of gram-negative bacteremia and ciprofloxacin resistance as patients transitioned from C1 to C2. Ciprofloxacin resistance was observed in 100% of the *E. coli* strains in C2, was rarely seen in *Pseudomonas* spp *(*1/4 in C1) and was not observed in the remaining *Enterobacteriaceae* (0/5 in C1 and 0/7 in C2). The preferential development of resistance among *E. coli* strains has been previously reported [18-20]. Cometta *et al.* noted that, among a cohort of patients receiving prophylactic fluoroquinolones, 28% of the *E. Coli* isolates were fluoroquinolone resistant while > 90% of the *P. aeruginosa* and *K. pneumoniae* isolates remained fluoroquinolone sensitive [18]. *Kern et al.* postulated the decreased virulence of fluoroquinolone resistant *P. aeruginosa* as one explanation for this phenomenon [20]. Prolonged exposure to subtherapeutic concentrations of antibiotics has been associated with the development of antibiotic resistance [21,22]. It is possible that decreased absorption of fluoroquinolones in the setting of chemotherapy-induced mucositis, may have prevented reaching the mutant prevention concentration [23,24] for *E. coli* in our cohort. We were unable to determine whether there was an emergence of resistance to other antibiotics, although the frequency of *E. coli* aminoglycoside resistance in C2 (4/6 cases) is concerning.

The effects of discontinuing prophylactic fluoroquinolones have been investigated in a number of studies. Reuter *et al*. prematurely stopped a trial of levofloxacin discontinuation due to a significant increase in infection-related mortality [25] and Kern *et al.* noted increased rates of bacteremia and a trend toward increased mortality during a period in which fluoroquinolone prophylaxis was discontinued [12]. A similar increase in bloodstream infections with fluoroquinolone susceptible isolates was seen when fluoroquinolone prophylaxis was discontinued by two Japanese groups although no effects on mortality were noted [26,27]. Thus, although the emergence of ciprofloxacin resistance organisms calls into question the value of ciprofloxacin prophylaxis, current evidence and IDSA guidelines support its use in high-risk patients with prolonged (≥ 7 days) and profound neutropenia (≤100 cells/mm^3^) [10].

The increased use of fluoroquinolone prophylaxis and indwelling CVC’s has led to gram-positive organisms as the main cause of bacteremia in immunocompromised patients [4,6,7,28]. In our cohort gram-positive organisms constituted 79% of the isolates during C1 and 70% in C2. As some of our patients were followed in community hospitals, we were unable to accurately determine which isolates were CVC related although we speculate that most CNST were of CVC origin. Consistent with previous reports [2,6,7,28,29], CNST and *Streptococcus spp.* were the most common gram positive organisms. The rate of CNST isolates was not statistically different between C1 and C2, however, *Streptococcus* isolates, particularly *S. viridans,* increased from C1 to C2 within the younger cohort, likely reflecting the cumulative effects of HiDAC chemotherapy [11,28,30-32].

As a high frequency of *S. viridans* infections is a well-documented effect associated with ciprofloxacin prophylaxis [12], patients within our cohort were administered amoxicillin during the neutropenic period. Surprisingly, however, all *Streptococcus* isolates with susceptibility data were found to be penicillin sensitive. Poor compliance with amoxicillin dosing or prior CVC colonization may provide a potential explanation for this observation. Although current IDSA guidelines recommend against the addition of gram-positive coverage to fluoroquinolone prophylaxis, studies have suggested that it may be beneficial in patients at high risk of streptococcal infections including those with severe neutropenia, oral mucositis, bone marrow transplantation and those receiving HiDAC-based chemotherapy [33]. From our data, it is unclear if the addition of amoxicillin alone resulted in any substantial benefit; delineation of this would require a prospective randomized study.

An important finding in our study, not previously described, is the increased rate of positive blood cultures in C2 in patients with previous positive cultures in C1. This effect was restricted to *Staphylococcus* spp. in younger patients and may reflect the prolonged use of indwelling catheters. The inadequate activity of ciprofloxacin and amoxicillin against these organisms likely further contributed to their increased pathogenicity [14,29]. It is possible that CVC replacement between consolidation cycles in patients previously positive for *Staphylococcus* spp. could reduce their recurrence rates, but this requires further study.

Our data have several limitations. This was a retrospective analysis conducted at a single tertiary care cancer center and it is unclear whether results are generalizable to other centers or with different regimens. Second, our rates of gram-positive bacteremia may have been overestimated as we could not always accurately differentiate whether isolates represented true bacteremia, CVC colonization or contamination. However, given the context of fever in high-risk neutropenic patients, all of whom were treated empirically with IV antibiotics, infection is more likely. Furthermore, as some patients were treated in community hospitals, we had incomplete information regarding antibiotic sensitivities.

## Conclusions

In summary, our findings emphasize the limitations of antibacterial prophylaxis of AML patients post-chemotherapy in the ambulatory setting, particularly in later cycles following prolonged use of fluoroquinolones and indwelling central venous catheters. Clinicians should be cognizant of the resulting increased predilection to fluoroquinolone-resistant *E. coli*, streptococcal spp. and recurrent CNST infections, and additional prophylactic measures should be considered in these patients.

## Competing interests

All authors declare that they have no competing interests.

## Authors’ contributions

LS performed the research and drafted the manuscript. CR revised the manuscript. EGA performed the statistical analysis. JMB participated in the study deign and revised the manuscript. All authors read and approved the final manuscript.

## Pre-publication history

The pre-publication history for this paper can be accessed here:

http://www.biomedcentral.com/1471-2334/13/284/prepub

## References

[B1] Tallman MS New strategies for the treatment of acute myeloid leukemia including antibodies and other novel agents Hematology Am Soc Hematol Educ Program 2005 143 150 16304372 10.1182/asheducation-2005.1.143

[B2] Girmenia C Alimena G Latagliata R Morano SG Celesti F Coppola L Spadea A Tosti S Mecarocci S D'Elia GM Out-patient management of acute myeloid leukemia after consolidation chemotherapy. Role of a hematologic emergency unit Haematologica 1999 84 9 814 819 10477455

[B3] Gillis S Dann EJ Rund D Selective discharge of patients with acute myeloid leukemia during chemotherapy-induced neutropenia Am J Hematol 1996 51 1 26 31 10.1002/(SICI)1096-8652(199601)51:1<26::AID-AJH5>3.0.CO;2-9 8571934

[B4] Eisele L Gunther F Ebeling P Nabring J Duhrsen U Durig J Outpatient management of acute myeloid leukemia after intensive consolidation chemotherapy is feasible and reduces hospital treatment costs Onkologie 2010 33 12 658 664 10.1159/000322209 21124036

[B5] Savoie ML Nevil TJ Song KW Forrest DL Hogge DE Nantel SH Shepherd JD Smith CA Sutherland HJ Toze CL Shifting to outpatient management of acute myeloid leukemia: a prospective experience Ann Oncol 2006 17 5 763 768 10.1093/annonc/mdl011 16497826

[B6] Moller T Nielsen OJ Welinder P Dunweber A Hjerming M Moser C Kjeldsen L Safe and feasible outpatient treatment following induction and consolidation chemotherapy for patients with acute leukaemia Eur J Haematol 2010 84 4 316 322 10.1111/j.1600-0609.2009.01397.x 20002732

[B7] Halim TY Song KW Barnett MJ Forrest DL Hogge DE Nantel SH Nevill TJ Shepherd JD Smith CA Sutherland HJ Positive impact of selective outpatient management of high-risk acute myelogenous leukemia on the incidence of septicemia Ann Oncol 2007 18 7 1246 1252 10.1093/annonc/mdm112 17442662

[B8] Allan DS Buckstein R Imrie KR Outpatient supportive care following chemotherapy for acute myeloblastic leukemia Leuk Lymphoma 2001 42 3 339 346 10.3109/10428190109064590 11699398

[B9] Ruiz-Arguelles GJ Apreza-Molina MG Aleman-Hoey DD Gomez-Almaguer D Marin-Lopez A Mercado-Diaz L Outpatient supportive therapy after induction to remission therapy in adult acute myelogenous leukaemia (AML) is feasible: a multicentre study Eur J Haematol 1995 54 1 18 20 7859871 10.1111/j.1600-0609.1995.tb01620.x

[B10] Freifeld AG Bow EJ Sepkowitz KA Boeckh MJ Ito JI Mullen CA Raad II Rolston KV Young JA Wingard JR Clinical practice guideline for the use of antimicrobial agents in neutropenic patients with cancer: 2010 Update by the Infectious Diseases Society of America Clin Infect Dis 2011 52 4 427 431 10.1093/cid/ciq147 21205990

[B11] Saini L Minden MD Schuh AC Yee KW Schimmer AD Gupta V Atenafu EG Murray C Nixon S Brandwein JM Feasibility of outpatient consolidation chemotherapy in older versus younger patients with acute myeloid leukemia Am J Hematol 2012 87 3 323 326 10.1002/ajh.22268 22213349

[B12] Kern WV Klose K Jellen-Ritter AS Oethinger M Bohnert J Kern P Reuter S von Baum H Marre R Fluoroquinolone resistance of Escherichia coli at a cancer center: epidemiologic evolution and effects of discontinuing prophylactic fluoroquinolone use in neutropenic patients with leukemia Eur J Clin Microbiol Infect Dis 2005 24 2 111 118 10.1007/s10096-005-1278-x 15714332

[B13] van Belkum A Goessens W van der Schee C Lemmens-den Toom N Vos MC Cornelissen J Lugtenburg E de Marie S Verbrugh H Lowenberg B Rapid emergence of ciprofloxacin-resistant enterobacteriaceae containing multiple gentamicin resistance-associated integrons in a Dutch hospital Emerg Infect Dis 2001 7 5 862 871 11747700 10.3201/eid0705.017515 PMC2631872

[B14] Bucaneve G Micozzi A Menichetti F Martino P Dionisi MS Martinelli G Allione B D'Antonio D Buelli M Nosari AM Levofloxacin to prevent bacterial infection in patients with cancer and neutropenia N Engl J Med 2005 353 10 977 987 10.1056/NEJMoa044097 16148283

[B15] Cullen M Steven N Billingham L Gaunt C Hastings M Simmonds P Stuart N Rea D Bower M Fernando I Antibacterial prophylaxis after chemotherapy for solid tumors and lymphomas N Engl J Med 2005 353 10 988 998 10.1056/NEJMoa050078 16148284

[B16] Leibovici L Paul M Cullen M Bucaneve G Gafter-Gvili A Fraser A Kern WV Antibiotic prophylaxis in neutropenic patients: new evidence, practical decisions Cancer 2006 107 8 1743 1751 10.1002/cncr.22205 16977651

[B17] Gafter-Gvili A Fraser A Paul M Leibovici L Meta-analysis: antibiotic prophylaxis reduces mortality in neutropenic patients Ann Intern Med 2005 142 12 Pt 1 979 995 15968013 10.7326/0003-4819-142-12_part_1-200506210-00008

[B18] Cometta A Calandra T Bille J Glauser MP Escherichia coli resistant to fluoroquinolones in patients with cancer and neutropenia N Engl J Med 1994 330 17 1240 1241 10.1056/NEJM199404283301717 8139646

[B19] Cattaneo C Quaresmini G Casari S Capucci MA Micheletti M Borlenghi E Signorini L Re A Carosi G Rossi G Recent changes in bacterial epidemiology and the emergence of fluoroquinolone-resistant Escherichia coli among patients with haematological malignancies: results of a prospective study on 823 patients at a single institution J Antimicrob Chemother 2008 61 3 721 728 10.1093/jac/dkm514 18218645

[B20] Kern WV Steib-Bauert M de With K Reuter S Bertz H Frank U von Baum H Fluoroquinolone consumption and resistance in haematology-oncology patients: ecological analysis in two university hospitals 1999–2002 J Antimicrob Chemother 2005 55 1 57 60 15574472 10.1093/jac/dkh510

[B21] Brown NM White LO Blundell EL Chown SR Slade RR MacGowan AP Reeves DS Absorption of oral ofloxacin after cytotoxic chemotherapy for haematological malignancy J Antimicrob Chemother 1993 32 1 117 122 10.1093/jac/32.1.117 8226402

[B22] Johnson EJ MacGowan AP Potter MN Stockley RJ White LO Slade RR Reeves DS Reduced absorption of oral ciprofloxacin after chemotherapy for haematological malignancy J Antimicrob Chemother 1990 25 5 837 842 10.1093/jac/25.5.837 2373666

[B23] Epstein BJ Gums JG Drlica K The changing face of antibiotic prescribing: the mutant selection window Ann Pharmacother 2004 38 10 1675 1682 10.1345/aph.1E041 15340128

[B24] Rybak MJ Pharmacodynamics: relation to antimicrobial resistance Am J Med 2006 119 6 Suppl 1 S37 44 discussion S62-70 16735150 10.1016/j.amjmed.2006.04.001

[B25] Reuter S Kern WV Sigge A Dohner H Marre R Kern P von Baum H Impact of fluoroquinolone prophylaxis on reduced infection-related mortality among patients with neutropenia and hematologic malignancies Clin Infect Dis 2005 40 8 1087 1093 10.1086/428732 15791505

[B26] Saito T Yoshioka S Iinuma Y Takakura S Fujihara N Ichinohe T Ishikawa T Uchiyama T Ichiyama S Effects on spectrum and susceptibility patterns of isolates causing bloodstream infection by restriction of fluoroquinolone prophylaxis in a hematology-oncology unit Eur J Clin Microbiol Infect Dis 2008 27 3 209 216 10.1007/s10096-007-0428-8 18058141

[B27] Chong Y Yakushiji H Ito Y Kamimura T Clinical impact of fluoroquinolone prophylaxis in neutropenic patients with hematological malignancies International journal of infectious diseases: IJID: official publication of the International Society for Infectious Diseases 2011 15 4 e277 281 10.1016/j.ijid.2010.12.010 21324723

[B28] Cannas G Pautas C Raffoux E Quesnel B Botton SD Revel TD Reman O Gardin C Elhamri M Boissel N Infectious complications in adult acute myeloid leukemia: analysis of the Acute Leukemia French Association-9802 prospective multicenter clinical trial Leuk Lymphoma 2012 53 6 1068 1076 10.3109/10428194.2011.636812 22145959

[B29] Madani TA Clinical infections and bloodstream isolates associated with fever in patients undergoing chemotherapy for acute myeloid leukemia Infection 2000 28 6 367 373 10.1007/s150100070007 11139156

[B30] Bakhshi S Singh P Swaroop C Outpatient consolidation chemotherapy in pediatric acute myeloid leukemia: a retrospective analysis Hematology 2009 14 5 255 260 10.1179/102453309X446144 19843379

[B31] Engelhard D Elishoov H Or R Naparstek E Nagler A Strauss N Cividalli G Aker M Ramu N Simhon A Cytosine arabinoside as a major risk factor for Streptococcus viridans septicemia following bone marrow transplantation: a 5-year prospective study Bone Marrow Transplant 1995 16 4 565 570 8528173

[B32] Okamoto Y Ribeiro RC Srivastava DK Shenep JL Pui CH Razzouk BI Viridans streptococcal sepsis: clinical features and complications in childhood acute myeloid leukemia J Pediatr Hematol Oncol 2003 25 9 696 703 10.1097/00043426-200309000-00005 12972804

[B33] Cruciani M Malena M Bosco O Nardi S Serpelloni G Mengoli C Reappraisal with meta-analysis of the addition of Gram-positive prophylaxis to fluoroquinolone in neutropenic patients J Clin Oncol 2003 21 22 4127 4137 10.1200/JCO.2003.01.234 14615441

